# Treatment of Central Nervous System Relapse in Acute Promyelocytic Leukemia by Venetoclax: A Case Report

**DOI:** 10.3389/fonc.2021.693670

**Published:** 2021-07-05

**Authors:** Xuzhao Zhang, Jinliang Chen, Weiqin Wang, Xian Li, Yanbin Tan, Xiaohong Zhang, Wenbin Qian

**Affiliations:** ^1^ Department of Hematology, The Second Affiliated Hospital of Zhejiang University School of Medicine, Hangzhou, China; ^2^ Center of Clinical Pharmacology, The Second Affiliated Hospital of Zhejiang University School of Medicine, Hangzhou, China; ^3^ Department of Radiology, The Second Affiliated Hospital of Zhejiang University School of Medicine, Hangzhou, China

**Keywords:** acute promyelocytic leukemia, central nervous system, relapse, venetoclax, CSF concentration

## Abstract

Extramedullary relapse of acute promyelocytic leukemia is a rare phenomenon and is associated with a poor prognosis, with the central nervous system being the most common site of relapse. The current treatments are still limited. Venetoclax, a selective inhibitor of BCL2, is a small molecule that can cross the blood-brain barrier and shows a potential efficacy in the treatment of chronic lymphocytic leukemia with central nervous system involvement. Although venetoclax has also been used in the treatment of acute myeloid leukemia in recent years, there are no reports of its use in the treatment of central nervous system relapse in acute promyelocytic leukemia. Here, we report a case of central nervous system relapse in acute promyelocytic leukemia that achieved complete remission after oral treatment with venetoclax. The presence of venetoclax in the patient’s CSF was confirmed by testing CSF and plasma by mass spectrometry. The concentration of venetoclax in CSF was approximately 1/300 of that in plasma trough concentration. The treatment experience in this case demonstrates the potential ability of venetoclax to treat of central nervous system relapse/involvement in acute promyelocytic leukemia, thus providing a new treatment option for this kind of patient.

## Introduction

Since the use of all-trans retinoic acid (ATRA) and arsenic trioxide (ATO) in the treatment of acute promyelocytic leukemia (APL), there have been extraordinary advances in the efficacy of APL treatment (1–4). Extramedullary relapse of APL is a rare phenomenon and is associated with a poor prognosis, with the central nervous system (CNS) being the most common site of relapse (5, 6). Median survival in patients with CNS relapse is shorter than that in patients with extracentral recurrences alone (5). Current treatments, including intrathecal chemotherapy, systemic chemotherapy with high-dose methotrexate (MTX) and/or cytarabine, radiation therapy and ATO (7), for CNS relapse in APL are still limited. Venetoclax, a selective inhibitor of BCL2, is a small molecule that can cross the blood-brain barrier and shows potential efficacy in the treatment of chronic lymphocytic leukemia (CLL) with CNS involvement (8). Although venetoclax has also been used in the treatment of acute myeloid leukemia (AML) in recent years (9, 10), there are no reports of its use in the treatment of CNS relapse in APL. Here, we report a case of CNS relapse in APL that achieved successful clearance of cerebrospinal fluid (CSF) APL cells after oral treatment with venetoclax. We hope to provide a new approach to the treatment of patients with CNS relapse in APL.

## Case Presentation

A 63-year-old male patient was admitted to our hospital with “diagnosed APL for more than 6 years and visual double vision for more than 2 months.” The patient had no family history and no specific social history. More than 6 years ago, this patient went to the local hospital for “recurrent gingival bleeding” and was diagnosed with APL in the low-risk group after providing the relevant examination. The patient received ARTA+ATO initial induction therapy, stopped ATO because of liver function impairment and switched to idarubicin and cytarabine therapy, which resulted in complete remission. Subsequently, the patient received intermittent consolidation and maintenance therapy with retinoic acid-arsenic acid. However, over the past 6 years, the patient had 4 relapses, obtaining remission after each relapse with salvage chemotherapy (details unknown). More than 2 months ago (APL in remission status), the patient had transient loss of consciousness without obvious inducement, which remitted spontaneously after a few moments. When he went to the local hospital, no typical epileptiform discharge was seen on electroencephalogram (EEG) and no obvious abnormality was seen on cranial magnetic resonance imaging (MRI). During the hospitalization, there was double vision, and a bone marrow smear revealed 1.57% promyelocytes. PML/RARα fusion gene test was negative. The lumbar puncture CSF test was not significantly abnormal. Cotton wool spot found on fundus examination was considered to be related to APL. During hospitalization, the patient had several epileptic-like seizures, and the symptoms improved slightly after neurology consultation with sodium valproate and mannitol to lower the cranial pressure. The patient was transferred to our hospital and was admitted to our department for further treatment.

**Figure 1:**
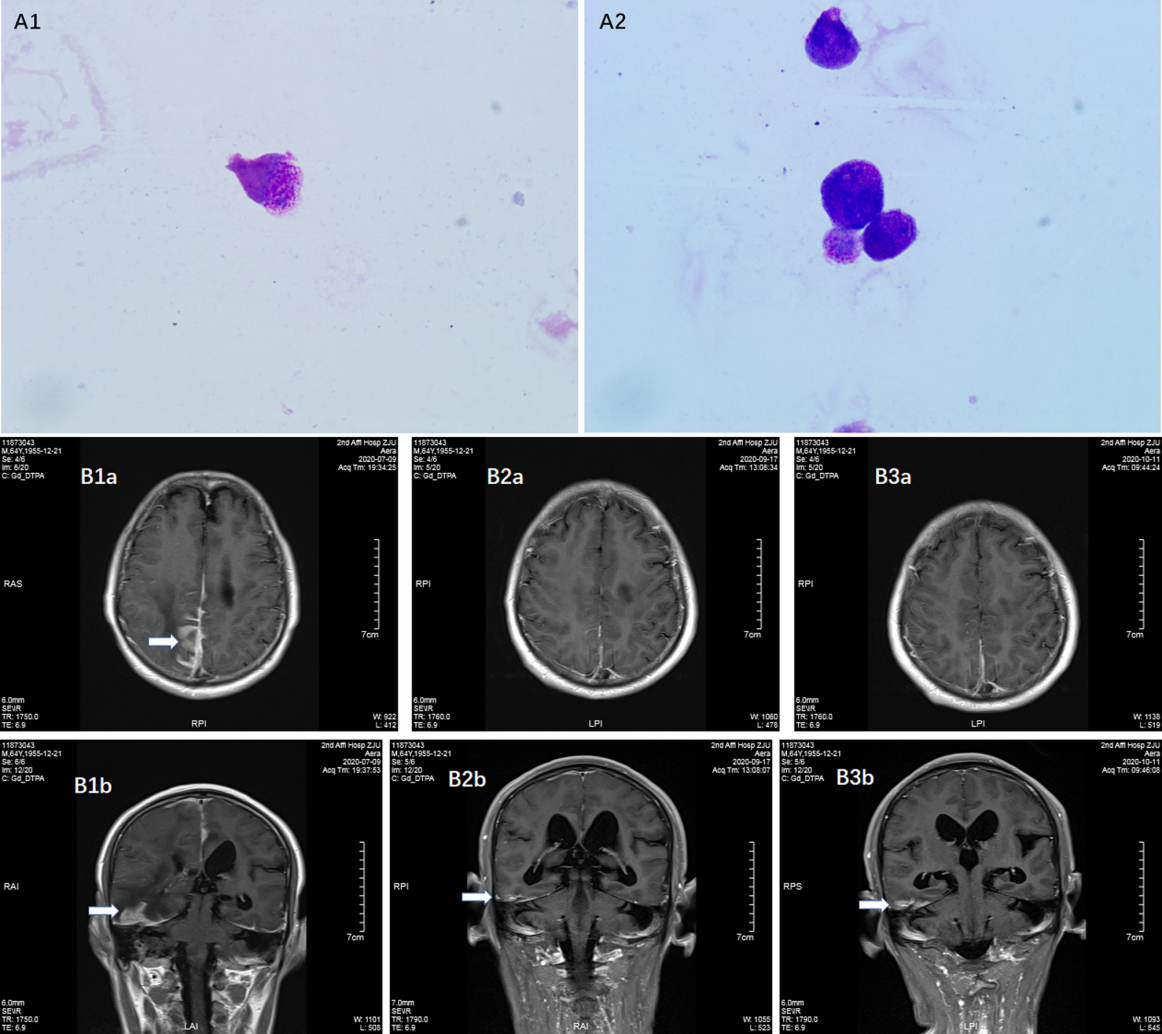
Detection of meningeal involvement by APL cells. **(A1–2)** Promyelocytic leukemia cells in CSF; **(B1a, 1b)** Contrast MRI before venetoclax treatment on July 9, 2020, **(B2a, 2b)** Contrast MRI during venetoclax treatment on September 17, 2020; **(B3a, 3b)** Contrast MRI during venetoclax treatment on November 11, 2020.

Laboratory tests: Complete blood count: white blood cell count 3 600/μL, hemoglobin 134 g/L, and platelet count 191 000/μL. The coagulation profile, serum biochemical tests, and tumor markers (including AFP, CEA, CA199, CA125, CA242, NSE, CA211, SCC and PSA) were all unremarkable. Bone marrow smear: no obvious leukemic cells were seen; the PML-RARα fusion gene was positive in bone marrow sample (215.49 copies, PML-RARα/ABL 0.001); bone marrow immunophenotyping: no obvious abnormal immune–phenotypes were seen. Peripheral blood was negative for the PML-RARα fusion gene. This patient subsequently underwent a lumbar puncture on July 16, 2019 with a CSF pressure of 300 mmH2O, 150 nucleated cells/μL (normal reference value <8/μL), 15% neutrophils, 60% lymph, and 25% mononuclear. CSF exfoliation cytology found abnormal promyelocytic leukemia cells (**Figures 1-A1, 2**). The PML-RARα fusion gene was positive in CSF (647 000 copies, PML-RARα/ABL 1.174). CSF flow cytometry testing: Abnormal promyelocytic leukemia cells account for approximately 77.475% of leukocytes. Diagnosis: 1. CNS leukemia (secondary to APL); 2. APL.

**Figure 2:**
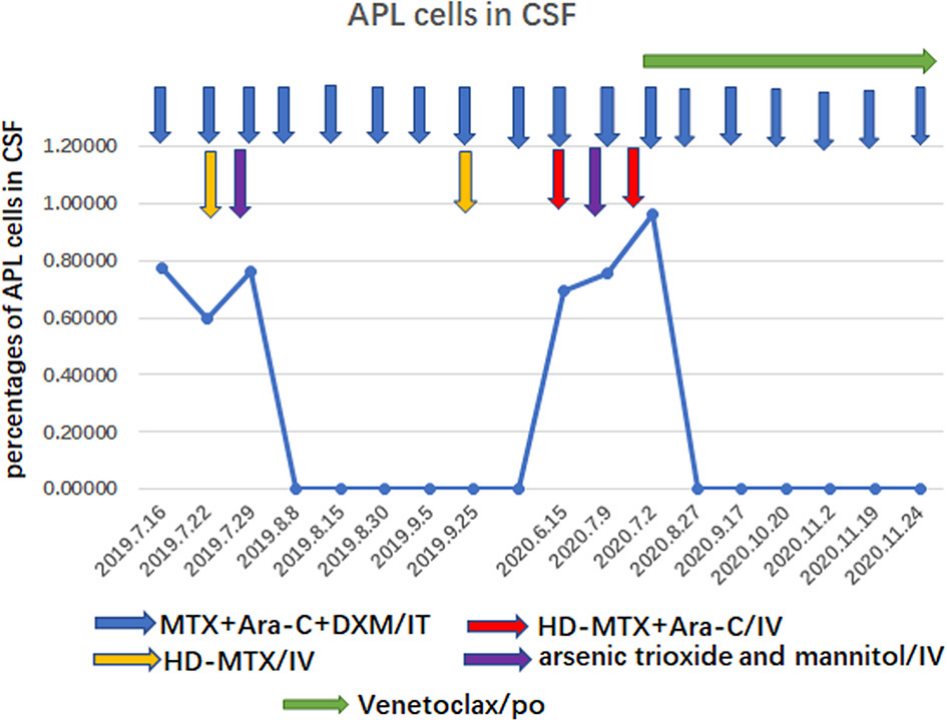
Trends of APL cells counts in CSF.

**Figure 3:**
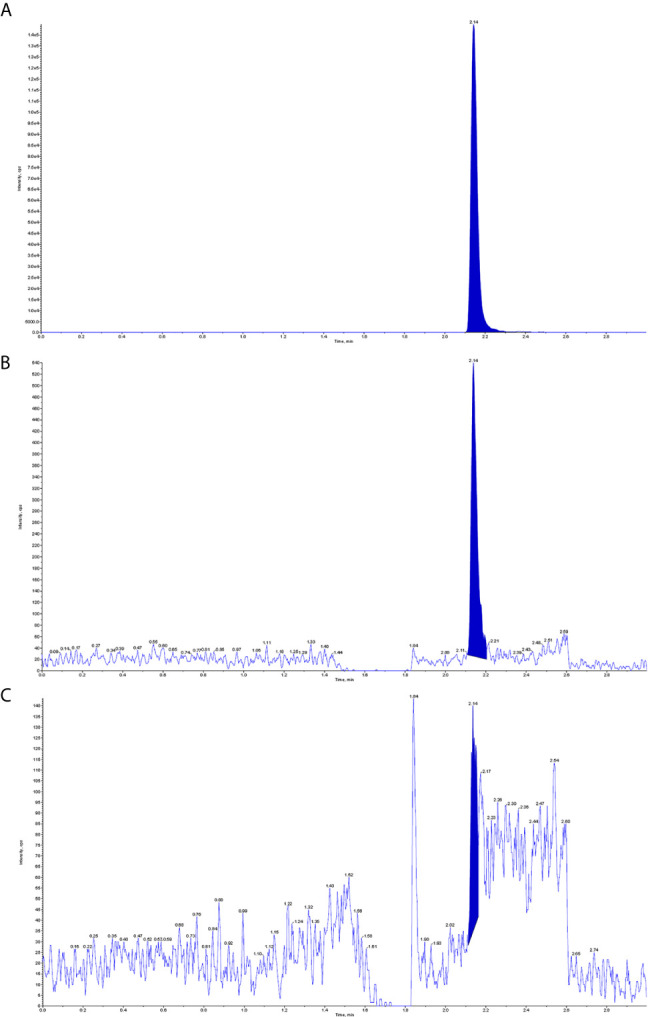
Detection of venetoclax in plasma and CSF by mass spectrometry. **(A)** Plasma venetoclax valley concentration; **(B)** Venetoclax concentration in the patient’s CSF; **(C)** Background concentration of venetoclax in the patient’s CSF before starting venetoclax.

The patient was treated with methotrexate, cytarabine and dexamethasone twice weekly intrathecal injections in combination with ATO and mannitol continuous intravenous alternating therapy for 30 days and the PML-RARα fusion gene was negative both in the CSF and bone marrow. The patient refused autologous hematopoietic stem cell transplantation (HSCT) consolidation for personal reasons. Subsequently, the patient received intermittent high-dose methotrexate and cytarabine combination chemotherapy and intrathecal injections of methotrexate, cytarabine and dexamethasone. Unfortunately, the patient has had a recurrence of head swelling since May 2020, and repeat lumbar puncture and cranial enhancement MRI suggested another intracranial relapse. Failure to induce remission again in this patient by triple intrathecal injection of methotrexate, cytarabine and dexamethasone in combination with ATO and mannitol continuous intravenous therapy. High-dose methotrexate in combination with medium-dose cytarabine also failed to work and resulted in severe myelosuppression. Then, the patient tried venetoclax oral treatment initially at a dosage of 100 mg once a day, and gradually increased to 400 mg once a day. After taking venetoclax for two weeks while continuing triple IT therapy, the patient’s head swelling was miraculously relieved, and a repeat lumbar puncture four weeks later showed complete disappearance of CSF leukemia cells and immunological remission of the bone marrow was also maintained (**Figure 2**). CSF leukemic cells continued to be negative by flow cytometry, but a repeat MRI of the brain still suggested possible localized meningeal involvement (**Figures 1-B1–3**). The presence of venetoclax in the patient’s CSF was confirmed by testing CSF and plasma specimens when the patient was on venetoclax for 4 weeks. The concentration of venetoclax in CSF was approximately 1/300 of that in the plasma trough concentration (**Figure 3**). This patient is still on ongoing venetoclax therapy and has maintained complete remission of CNS for 8 months (**Figure 2**). He is still undergoing regular follow-up. To the best of our knowledge, this is the first case of venetoclax applied to CNS relapse of APL.

## Discussion

CNS relapse is a low incidence event in APL but is a factor significantly associated with poor prognosis. Similar to the present case, CNS relapse in APL can occur several years after achieving CR and is often difficult to manage. Although there are very few cohort studies to try new treatment strategies for CNS relapse in APL, current experience in the treatment of this situation is overwhelmingly based on case reports. Therefore, finding new therapeutic measures is an urgent challenge that needs to be addressed.

The Bcl-2 inhibitor venetoclax is a small molecule that has shown promising therapeutic effects in AML (9, 10) and lymphatic malignancies (11–13). There are also few case reports of the use of venetoclax in cases of CNS involvement in CLL (8). In vitro experiments revealed that the APL cell line NB4 has good sensitivity to venetoclax (14). However, to date there have been no reports of venetoclax for the treatment of CNS involvement/relapse in APL.

This is a refractory case of APL with CNS relapse, which did not respond well to intrathecal injection and high-dose MTX-based regimens. In this case, venetoclax showed excellent anti-meningeal APL efficacy and was well tolerated with ongoing triple IT therapy. A recent study found that venetoclax can cross the blood-brain barrier and exert antimembranous CLL activity, and its CSF concentration is approximately 1/1000 of the plasma concentration (8). In this study, we also demonstrated that the presence of venetoclax in CSF can be detected by mass spectrometry, and the concentration in CSF is approximately 1/300 of the plasma trough concentration. This finding could explain the effectiveness of venetoclax for meningeal APL. However, the lack of controls limits the absolute quantification of venetoclax concentrations in plasma and CSF.

Although this patient’s brain MRI still suggested possible localized meningeal involvement, the experience of this case offers a new option for the treatment strategy of CNS involvement/relapse in APL as well as other kinds of AML. However, the treatment experience in this case also suggests that single agent venetoclax therapy still seems to be inadequate and more effective combination therapy options still need to be explored to improve treatment efficacy. This case is only an empirical report of a single case, and the efficacy of venetoclax in CNS APL involvement will need to be confirmed in a large clinical study.

## Data Availability Statement

The original contributions presented in the study are included in the article/supplementary material. Further inquiries can be directed to the corresponding authors.

## Ethics Statement

The studies involving human participants were reviewed and approved by the ethics committee of the Second Affiliated Hospital of Zhejiang University School of Medicine. The patients/participants provided their written informed consent to participate in this study. Written informed consent was obtained from the individual(s) for the publication of any potentially identifiable images or data included in this article.

## Author Contributions

XuZ and WQ designed the study. XuZ, WW, XL, and XiZ collected the data. WW performed the cytology analysis of APL cells in CSF. JC performed the analysis of venetoclax in plasma and CSF. YT performed the MRI analysis. XuZ and WQ wrote the manuscript. All authors contributed to the article and approved the submitted version.

## Funding

This work was supported by a grant from the National Natural Science Foundation of China (81000895 to XuZ) and a grant from Zhejiang Provincial Natural Science Foundation of China (LY14H160032 to XuZ).

## Conflict of Interest

The authors declare that the research was conducted in the absence of any commercial or financial relationships that could be construed as a potential conflict of interest.

## Abbreviations

APL, acute promyelocytic leukemia; CNS, central nervous system; AML, acute myeloid leukemia; ATRA, all-trans retinoic acid; ATO, arsenic trioxide; MTX, methotrexate; CLL, chronic lymphocytic leukemia; CSF, cerebrospinal fluid; EEG, electroencephalogram; MRI, magnetic resonance imaging; HSCT, hematopoietic stem cell transplantation.
